# Non-neoplasic and non-syndromic palatal perforations. Presentation of 5 cases and systematic review of the literature

**DOI:** 10.4317/jced.58714

**Published:** 2021-09-01

**Authors:** Brenda-Daniela Ortega-Hidalgo, Karen Monge, Vania Pérez, María del Carmen Villanueva-Vilchis, Luis-Alberto Gaitán-Cepeda

**Affiliations:** 1 Dental student, Dental School, National Autonomous University of Mexico, Mexico City, Mexico; 2 Dental student, Dental School, University of Sonora, Hermosillo, Sonora, México; 3 Full time Professor of Oral Public Health Department, National School of Superior Studies – León, León, Guanajuato, México; 4 Full time Professor of Department of Oral Pathology and Oral Medicine, Graduate and Research Division, Dental School, National Autonomous University of Mexico. Mexico City, Mexico

## Abstract

**Background:**

Palatal perforations not associated with syndromes or neoplasms are rare lesions whose frequency has increased recently. However, their clinical and demographic characteristics have not been fully described. Therefore, this report aimed to establish the demographic and clinical characteristics of patients with non-syndromic and non-neoplastic palatal perforations.

**Material and Methods:**

The file of an oral medicine teaching clinic from January 2004 to December 2018 was reviewed to identify and isolate all cases with a diagnosis of palatal perforation. Cases with a diagnosis of palatal perforation related to congenital alteration, syndrome, or neoplasia were excluded. Age, sex, medical history, and diagnosis were obtained from the clinical history. In addition, a systematic review of the literature was performed using a PICO strategy. MEDLINE electronic databases from January 1990 to December 2018 were systematically reviewed using the combination of keywords with Boolean terms “OR” (palatal perforation, destruction of the palate) and “AND” (drugs, cocaine, mycosis, syphilis, mucormycosis, tuberculosis, trauma). The PRISMA guide was used to identify the different results of the literature search and article selection process. Case reports and case series were included.

**Results:**

Five cases of non-syndromic, non-neoplastic palatal perforations were identified. All cases were male with a mean age of 42 years. Two cases were related to cocaine use, 2 cases were caused by mucormycosis, and one case by trauma. As for the systematic literature review, 51 non-neoplastic and non-syndromic cases were collected. The cases showed a male predominance, with a mean age of 41 years. The most frequent etiology was chronic cocaine use followed by mucormycosis.

**Conclusions:**

Since cocaine use and type II Diabetes Mellitus, risk factors related to non-syndromic and non-neoplastic palatal perforations, have shown a worldwide increase, the clinician should be alert to make an early diagnosis and initiate appropriate treatment.

** Key words:**Palatal perforation, cocaine-induced, mucormycosis, mycotic infection, drug users.

## Introduction

Palatal perforations are rare lesions. Their most common etiology includes genetic and congenital alterations (1), followed by neoplasms (2). On the other hand, non-neoplastic and non-syndromic palatal perforations are much less frequent lesions. The most frequent etiological factors of non-neoplastic and non-syndromic palatal perforation are fungal infections caused mainly by the genus Mucor (3), and recreational drug use (cocaine) (4).

Palatal perforations have a significant impact on the quality of life and in some cases compromise the life of the patient who suffers from it. The correction of palatal perforation and the rehabilitation of patients suffering from them is complex, costly, and multidisciplinary. It involves general dentists, oral pathologists, plastic surgeons, maxillofacial surgeons, maxillofacial prosthetists, dental rehabilitators, psychologists, and speech therapists. Since patients with palatal perforations may request professional consultation in the general dental practice, the general dentist should be alert to establish a correct diagnosis to initiate treatment and rehabilitation as soon as possible and, consequently, improve the quality of life of these patients.

The clinical-demographic profiles of patients with non-neoplastic and non-syndromic palatal perforations are not well established. Therefore, the main objective of this paper was to establish the clinical and demographic characteristics of patients suffering non-syndromic and non-neoplastic palatal perforations, using a case series of patients coming from an oral medicine teaching clinic and a systematic review of the scientific literature on non-neoplastic and non-syndromic palatal perforations.

## Material and Methods

-Cases.

The archive of the Oral Medicine Clinic, Department of Oral Medicine and Oral Pathology of the Dental School, National Autonomous University of Mexico, Mexico, was reviewed from January 2009 to December 2018 to identify and isolate all cases with a diagnosis of palate perforation. Those cases of palatine perforations whose etiology included congenital alterations, syndrome, or neoplasia were excluded. To be included in the present study, the medical record of the selected cases should have the following data: age, gender, etiology, and diagnosis. For research purposes, a palatal perforation was defined as the loss of the component tissues of the palate involved the entire thickness of the palate, soft or hard, in such a way that an oral-nasal or oral-nasopharyngeal communication was presented.

-Systematic review of the literature.

Electronic databases OVID, PubMed, Scopus, and Web of Science were systematically reviewed for publications that present data on palatal perforations. The structured questions formulated for this search were as follows: What are the clinical-demographic characteristics of patients suffering non-syndromic or non-neoplastic palatal perforations and what are the main causes of non-syndromic and non-neoplastic palatal perforations? Then, following the PICO [Patient Problem, (or Population), Intervention, Comparison (or Control), Outcome] strategy for this search, using Boolean operators, we combined by “AND” and separated by “OR” the following keywords: “Palatal perforation”, “Palate perforation”, “midline destructive”, “drugs”, “cocaine”, “mycosis”, “syphilis, “mucormycosis”, “tuberculosis”, “traumatic”.

The resulting articles strictly fulfilled the search inclusion criteria to be selected; otherwise, they were excluded. In the present study, the articles eligible for inclusion were those that in the content of the title or the abstract had the phrase “palatal perforation (hard or soft)”. Other inclusion criteria were: (i) articles published from January 1990 to December 2018; (ii) articles published in English or Spanish languages, (iii) case reports or reports of case series. The exclusion criteria were: (i) case reports of palatal perforation with a diagnosis of any syndrome, congenital dysmorphology, or malignant neoplasia; (ii) case reports without demographical data o with incomplete data, (iii) case reports without a diagnosis; (iv) articles published outside the established time frame. The title and the abstract were reviewed to select articles for further reading and analysis, to avoid misleading data. Afterward, the papers were included and classified according to their diagnosis and geographical distribution.

After eliminating duplicates, the authors, independently, evaluated the titles, abstracts, and full text of the selected articles, to agree on the articles chosen and included according to the criteria. The following data were obtained from each of the selected articles: author(s), year of publication, country of origin of the patient, age, gender, personal history, medical history, and clinical characteristics of palatal perforation. An ex profeso database was then built using a spreadsheet (Excel Microsoft ®). For statistical purposes, a chi square test for nominal variables and t student test for ordinal variables were done to establish bivariate associations. In both cases, the significance level was established at 95% (*p*<0.05).

## Results

-Cases 

Five cases of non-syndromic and non-neoplastic palatal perforations were included. Regarding their etiology, two were related to chronic use of cocaine, 2 cases (40%) were caused by mucormycosis, and one case (20%) by trauma. All patients were male with an age range from third to seventh decades of life, and an age average of 42 years.

**Figure 1:**
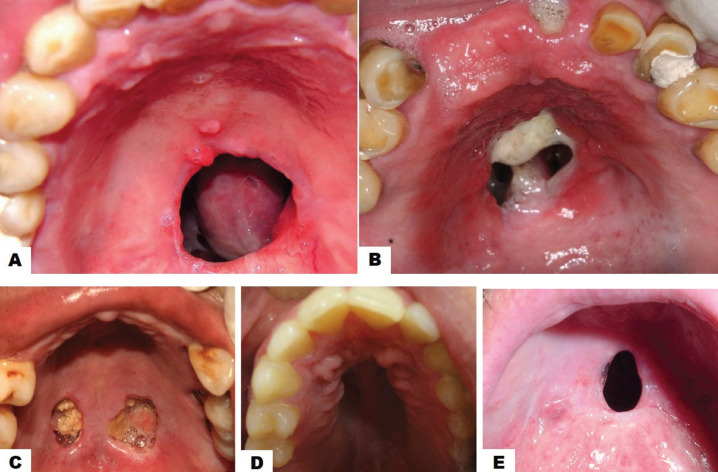
Clinical aspects of 5 cases of non-syndromic and non-neoplastic palate perforations. A. Palatal perforation of a circular shape in a 69-year diabetic man with a previous diagnosis of palatal mucormycosis (Case 1). Notice the oval perforation located in the middle line of the hard palate. B. Palatal perforation in a 56-year male, consuetudinary cocaine consumer. Notice the oronasal communication of circular shape and 4 cm in diameter. Observes the surrounding reddish halo corresponding to erythematous candidiasis and caseous necrosis at their bottom (Case 2). C. A 58-year uncontrolled diabetic male patient and with a diagnosis of mucormycosis show two oval-shaped ulcers sited at both sides of the midline of the hard palate. The bottom of both ulcers showed caseous necrosis (Case 3).D. Palatal perforation in a 25-year male, consuetudinary cocaine consumer. Notice the well-defined circularly palatal perforation (Case 4).

Case 1: A 69-year-old man suffering from diabetes mellitus 2 was referred to the Oral Medicine Clinic by the Maxillofacial Prosthetics Clinic of the same unit due to the presence of a palate perforation related to a previously treated clinical event of mucormycosis. To the clinical inspection, it was observed an oro-nasal communication of oval shape, located in the middle line of the hard palate. The perforation was extended from the mesial zone of the canines to the distal zone of the second bicuspids. A smear was performed on mucosal limiting the perforation, which was seeded in selective culture media. The culture was negative for mucor, so the patient was counter-referred to the clinic of maxillofacial prosthesis to continue his rehabilitation (Fig. 1A).

Case 2: 56-year-old male, with no personal pathological history, with a family history of cancer, was referred to the Oral Medicine Clinic by the admission service of the same institution. Clinical examination showed an oronasal communication associated with cocaine use. The circular perforation, approximately 4 cm in diameter, was ubicated in the hard palate. Around the perforation, there was a reddish halo corresponding to erythematous candidiasis. At the bottom of the perforation was observed caseous necrosis. Antifungal treatment for candidiasis was instituted and the patient was subsequently referred to the maxillofacial prosthodontics clinic for rehabilitation (Fig. 1B).

Case 3: A 58-year-old male patient with a history of uncontrolled type 2 diabetes was referred to the oral medicine clinic by his private dentist due to the presence of two deep ulcers at both sides of the midline of the hard palate. The patient reported that occasionally self-administering metformin/glibenclamide. At the clinical inspection was observed two oval-shaped ulcers sited at both sides of the midline of the hard palate. The bottom of both ulcers showed caseous necrosis. A smear, with sterile hyssop, was performed from the bottom of the lesion to be seed in selective culture media. The culture and the microscopic observation of the smear confirmed the diagnosis of mucormycosis. Antimycotic treatment was initiated versus mucormycosis, and then the patient was referred to the department of maxillofacial surgery for surgical treatment (debridement). Subsequent debridement of ulcers revealed oral-nasal communication in the ulcer on the left side of the hard palate. Eventually, the patient was referred to the clinic of maxillofacial prosthesis for their rehabilitation (Fig. 1C).

Case 4. A 25-year-old male, with antecedents of chronic alcoholism and consumption of various recreational drugs, was referred from the admission service from the same institution. At the clinical inspection, it was observed a well-defined circularly palatal perforation, sized 3 cm, located in the middle line of the hard palate. The patient reported the use continuous of cocaine. The patient stopped attending their appointments so the case could not be followed up (Fig. 1D).

Case 5: A 60-years-old male, without pathological antecedents, was referred to the oral medicine clinic by their private dentist because he presents a palatal perforation on the midline in the junction of the hard and soft palate. The palatal perforation was well defined, of an oval shape, and 2cm in diameter. In the anamnesis, the patient reported that two years before his auscultation, he scratched with his fingernails a wart, of unknown etiology, located on the soft palate. After the scratching, perforation of the soft palate started. The patient was referred to the maxillofacial prosthesis clinic for rehabilitation (Fig. 1E).

-Systematic Review of scientific literature.

**Figure 2:**
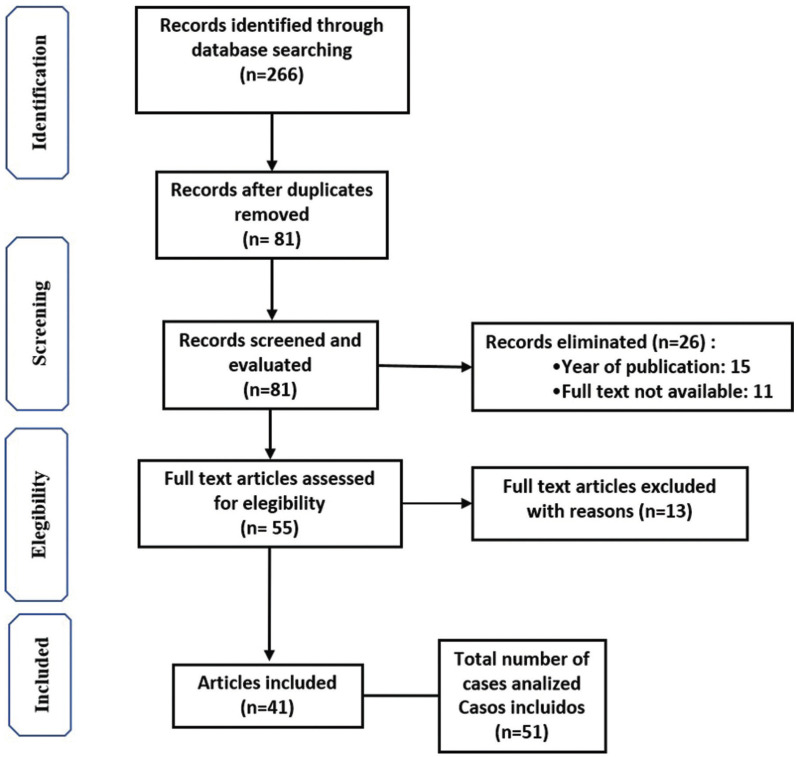
PRISMA Flow diagram.

The systematic review of the literature revealed 266 articles related to palatal perforations. Posterior to elimination because of their etiology of the cases, neoplasms, or syndromes, or because they were reviews of the literature, 81 articles were selected. From these, 26 were eliminated because they were outer of the frame time or because the full text was not available. In such a way 55 articles were analysed. From these 14 were excluded because of incomplete demographical or clinical data. In such a way 41 articles were included in the analysis. Because in some papers two or more cases were reported, the total of cases includes in the present study was 51 (5-44) (Fig. 2). Then, the five cases coming from our oral medicine clinic were added to the 51 cases found in the literature, therefore a total of 56 cases were analysed.

From 56 cases, 32 were males (57.1%) and 24 females (42.9%); the mean age of total of cases was 39.3 years (Standard deviation ±16.7) (Table 1). The use of recreative drugs, specifical cocaine, was the most frequent etiology with 25 cases representing 51.7% of whole cases, followed by infections with 17 cases (30.3%). Trauma was the etiology in 8 cases (14.2%). The distribution of total of cases regarding gender and etiology is shown in Table 2.

**Table 1:**
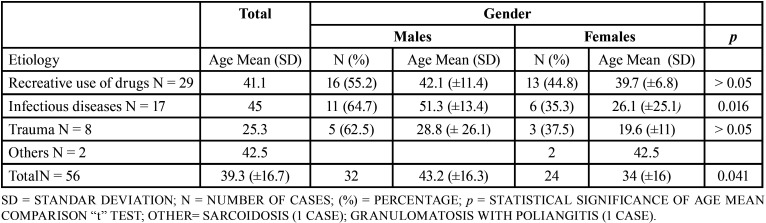
Comparison of age and gender of 56 cases of non-syndromic and non-neoplastic palatal perforations regarding etiology.

**Table 2:**
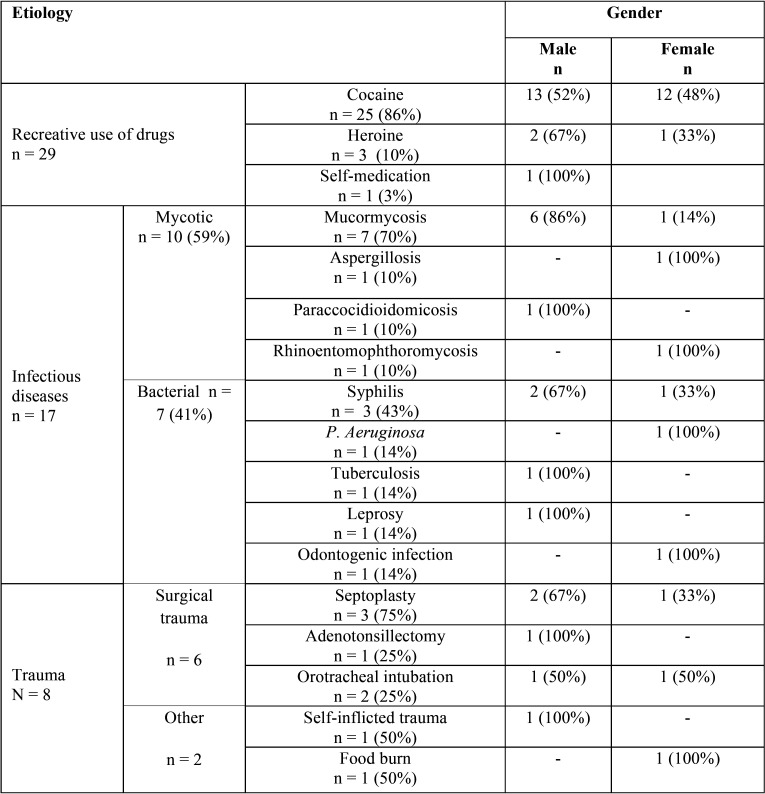
Distribution of 56 cases of non-syndromic and non-neoplastic palatal perforations regarding gender and etiology.

Concerning palatal perforations caused by the consume of recreational drugs, the most frequent reported drug was cocaine follows by heroin (Table 2). The age average of all reported cases of palatal perforations related to use of drugs was 41.1 years. Regarding gender, from 29 cases, 55.1% were males. Most of cases coming from the United States of America followed by Spain and Brazil (Table 3).

Seventeen cases (11 male; 6 female) of palatal perforation were caused by infectious processes, (Table 1). The age average of these patients was 45 years, The most prevalent infection was the fungal one, principally mucormycosis follows by Aspergillosis, Paracoccidiomycosis, and Rhinoenteromycosis. Bacterial infections were less prevalent, being syphilis the most frequent followed by leprosy and tuberculosis. Regarding the geographical distribution, most cases coming from India, followed by England, Mexico, and Turkey (Table 3).

**Table 3:**
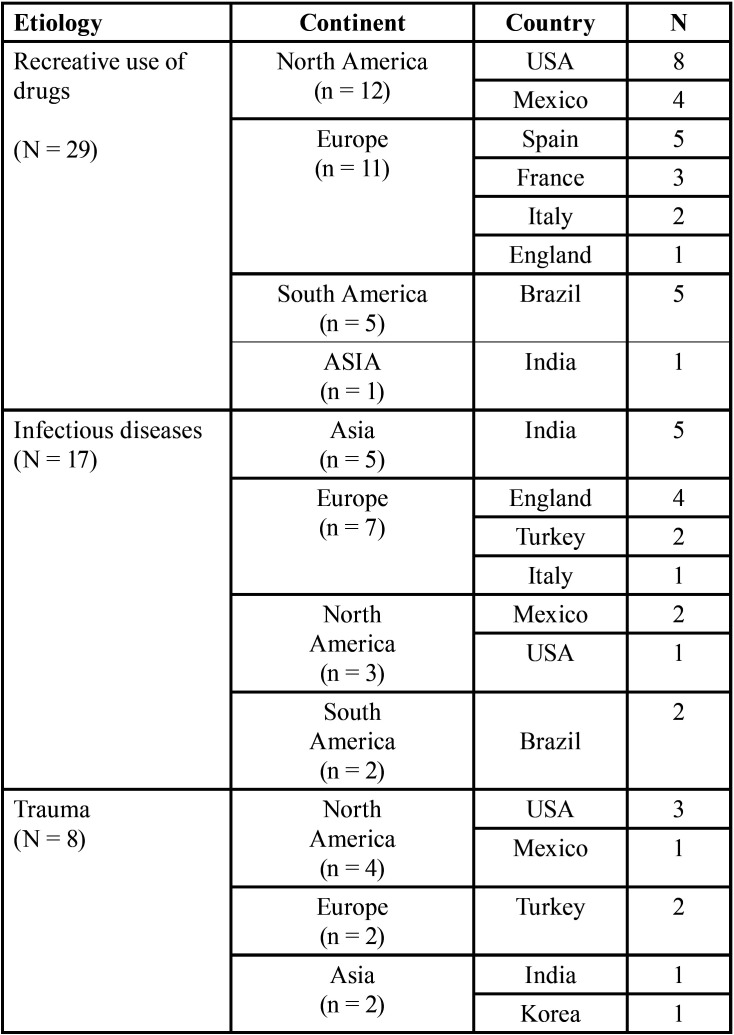
Geographical distribution of 56 cases of non-syndromic and non-neoplastic palatal perforations regarding gender and etiology.

We identified 8 cases of palatal perforations related to trauma, 5 of them were male. The age average was 25.3 years, significantly less than other etiologies. The most prevalent traumatic act reported were surgical events, followed by orotracheal intubation (Table 2).

Other etiologic factors were sarcoidosis, granulomatosis and, polyangiitis, with one case each.

Regarding statistical analysis, we did not find a statistical difference between gender distribution concerning etiology, *p* >0.05 in all cases. On the other hand, we found statistical differences between the age average of patients suffering palatal perforations related to use of recreative drugs and patients with traumatic etiology, 41.1 years vs 25.3 years respectively (*p* 0.004), and between age average of males (43.2 years) versus females (34 years) (*p* 0.041) (Table 1). In cases of palatal perforations caused by infectious diseases, males were older than females, 51.3 years (SD ±13.4) versus 26.1 years (SD ± 25.1), being this difference statistically significant (*p* 0.016) (Table 1).

## Discussion

The present study assesses the demographical and clinical characteristics of non-syndromic and non-neoplastic palatal perforations about their etiology and geographical distribution. Our results show that the most frequent cause of this kind of palate perforation is cocaine use. Cocaine is the third substance most consumed globally, surpassed only by cannabis and opioid (45). Since the 80s of the last century, it was reported an increase in the number of cocaine users worldwide (46). In the United States of America, in 2002-2003, 1.2 million inhabitants had consumed cocaine at least one time in their lives. For the period 2012-2013 the prevalence of use had almost doubled (47). In such a way that in 2015 there were approximately 3.9 million cases globally (45). North America region, comprising the USA and Canada had one of the most prevalent rates of cocaine dependence 301.2 per 100 000 people. (45) This data may justify our finding showed to America as the country where more cases of palatal perforation related to cocaine consumption were reported.

On the other hand, in Europa cocaine use is rising. In 2014 it was estimated that about 2.3 million young adults (from 15 to 34 years of age) consumed this substance, being Spain, Italy, and the United Kingdom the countries with the highest consumption (48). For Latin America in 2014 approximately 2.2% population over 14 years of age in Brazil has used cocaine in some way, and that approximately 2.4 million people, consume it in an inhaled form (49). This data supports our findings that both in our cases and in those identified in the systematic review, the most frequent etiology of palatal perforations is the use of cocaine, specifically in North America. In Mexico, our country, the consumption of cocaine is frequent too. The National Survey of Drug, Alcohol, and Tobacco Use in Mexico of 2016 and 2017 reported that almost 10% of respondents have used illegal drugs at some point in their lives, being the cannabis and cocaine the most frequents. Male is the most prevalent gender (50). In the cases presented related to cocaine use we obtained an age average of 42 years; this data coincides with the data reported as the age of greater exposure to drugs (50). Regardless of geographic distribution, the above data highlight the enormous number of potential patients who could develop a palatal perforation.

A similar possibility could be identified in palatal perforations related to fungal infections. Mucormycosis, which represents 40% of reported cases, is strongly linked to a pre-existing systemic condition and immunodeficiency response, specifically, mucormycosis has been related to non-controlled type II diabetes mellitus. Mucormycosis destroys the palate and facial bones surrounding the ocular cavity, paranasal sinuses, and nasal septum generating oroantral communications (4). According to the “National survey on health and nutrition, 2012” (México), in Mexico type II diabetes mellitus ranks first in mortality and morbidity. It is estimated that about 4 million people in Mexico suffer this condition and, of special importance to this report, over 80% of these patients receive treatment (51). These data are relevant because suggest that 20% of diabetic patients in Mexico have not under treatment and therefore there are 800,000 potential patients at risk to suffers infections such as mucormycosis. This fact could justify why mucormycosis was one of the most frequent etiology for the non-syndromic and non-neoplasic palatal perforations. Therefore, a general dentist or dental specialist may be the first professional to observe and attend to that recipient of patients with this systemic condition and with the propitious systemic conditions to develop opportunistic infections. Another type of zygomycosis has been reported in India caused by Conidiobolus coronatusson causing rhinoentomycetophthoromycosis, found only in tropical rainforests making this disease rare (38). It was also found a case of palatal perforation caused by paracoccidioidomycosis reported in Brazil, whose causal agent is the fungus Paracoccidioides brasiliensis which is an endemic species of South America, making less the possibility of accidental inhalation and inoculation of this fungus species, besides that this disease is rarely associated with the destruction of bones, such as the palatine bone (6).

## Conclusions

Due to the increase in the population susceptible to developing palatal perforations, the general dentist should be alert to identify palatal perforations, determine their etiology and choose the corresponding treatment or refer the patient to a specialist in oral medicine. The management of these perforations is multidisciplinary and includes general dentists, oral pathologists, maxillofacial surgeons, maxillofacial prosthodontists, speech therapists, psychologists, among others.
